# Targeting VEGF using Bevacizumab attenuates sepsis-induced liver injury in a mouse model of cecal ligation and puncture

**DOI:** 10.25122/jml-2023-0064

**Published:** 2023-10

**Authors:** Aula Zaini, Hayder Edrees Jawad, Najah Rayish Hadi

**Affiliations:** 1 Department of Pharmacology and Therapeutics, Faculty of Medicine, University of Kufa, Najaf, Iraq; 2 Karbala Health Directorate, Alhindiyah General Hospital, Karbala, Iraq

**Keywords:** VEGF, Bevacizumab, sepsis-induced liver injury, mouse model

## Abstract

Sepsis, a life-threatening condition resulting from an uncontrolled host response to infection, often leads to severe liver damage and remains a significant cause of mortality in critically ill patients despite advances in antibiotic therapy and resuscitation. Bevacizumab, a neutralizing antibody targeting vascular endothelial growth factor (VEGF), is approved for treating certain cancers. However, its potential impact on sepsis-related liver injury is not well understood. This study aimed to explore the potential hepatoprotective effect of Bevacizumab on sepsis-induced liver injury. Twenty-four mice were divided into four groups: a sham group subjected to a midline incision only, a cecal ligation and puncture induction (CLP) group, a vehicle-treated group that received a vehicle one hour before CLP induction, and a Bevacizumab-treated group that received Bevacizumab one hour before CLP induction. Blood samples were collected, and angiopoietin-2 (ANGPT2), alanine transaminase (ALT), and aspartate transaminase (AST) serum levels were measured. Liver tissue homogenates were analyzed for IL-6, TNFα, intracellular adhesion molecule (ICAM-1), macrophage inhibitory factor (MIF), vascular endothelial growth factor (VEGF), F2-isoprostane, and caspase-11 levels. A histological examination was performed to assess the extent of liver damage. Mice exposed to CLP had high levels of the biomarkers mentioned above with a high degree of liver injury compared to the sham group. In contrast, treatment with Bevacizumab notably reduced these markers and mitigated liver damage. In conclusion, Bevacizumab may be a protective agent against sepsis-induced liver injury.

## INTRODUCTION

Sepsis is an uncontrolled immune response of the host to infection, toxins, and trauma, having a considerable impact on the population worldwide and resulting in a high percentage of morbidity and mortality [1]. While multiple molecular mechanisms contribute to the pathogenesis of sepsis, few studies have investigated the main mechanisms underpinning organ damage as a consequence of sepsis [2]. Sepsis affects over 30% of the global population, resulting in around six million sepsis-associated deaths [3]. Future research in this field would greatly help reduce the deterioration of sepsis-related organ damage.

Over four decades ago, vascular endothelial growth factor (VEGF) was identified as an endothelial permeability factor. Later, its diverse roles, including its involvement in endothelial cell migration, proliferation, and survival, have been recognized. [4]. VEGF exerts its biological effects by binding to two receptors, VEGFR-1 and VEGFR-2, with different signaling properties [5]. Preclinical studies reported that the levels of VEGF and VEGFR-1 are elevated during sepsis, suggesting that VEGF aggravates sepsis and mediates morbidity and mortality [6]. Neutralizing VEGF could be a therapeutic opportunity that could contribute to reducing organ damage. Bevacizumab is an anti-VEGF monoclonal antibody that selectively binds VEGF-A isoforms and subsequently blocks the interaction between VEGF-A and their VEGF receptors on the surface of endothelial cells [7]. It has received the Food and Drug Administration (FDA) approval to treat patients with recurrent or metastatic cervical cancer in combination with paclitaxel and cisplatin or topotecan [8]. Up to now, there has been limited research concerning the effect of Bevacizumab on sepsis, and only a few studies have investigated the role of Bevacizumab as a therapeutic opportunity targeting VEGF molecules that are critical in the pathogenesis of sepsis. Some studies highlighted the role of Bevacizumab as an anti-VEGF agent in sepsis models and found that it attenuates mediators of inflammatory response and increases the survival probability [2]. Therefore, the current study aimed to assess the potential effect of Bevacizumab on sepsis-induced liver injury using cecal ligation and puncture (CLP).

## MATERIAL AND METHODS

All experiments were conducted at the Department of Pharmacology and Therapeutics and the Middle Euphrates Unit for Cancer Research, University of Kufa, Iraq. Animals were kept in the animal house at the University of Kufa and were allowed free access to food and water. Bevacizumab and the protease inhibitor cocktail were obtained from Med Chem Express, USA. Ketamine vials of 50 (100 mg/ml) were obtained from Alfasan, Holland, and Xylazain vials of 25 ml (20 mg/ml) from Arendonk, Belgium. Enzyme-linked immunosorbent assay (ELISA) kits for interleukin-6 (IL-6), tumor necrosis factor-alpha (TNF-α), macrophage inhibitory factor (MIF), vascular endothelial growth factor (VEGF), intracellular adhesion molecule (ICAM-1), angiopoietin-2 (ANGPT2), F2-isoprostane, caspase-11 were obtained from Bioassay Technology Laboratory, China. Alanine transaminase (ALT) and aspartate transaminase (AST) were purchased from Roche Diagnostics, Germany.

### Study groups

Twenty-four adult male albino Swiss mice, aged 8-12 weeks and weighing between 25 and 35 g, were divided into four groups, each consisting of six mice. The sham group was anesthetized and subjected to a laparotomy without performing cecal ligation and puncture (CLP). The CLP group was subjected to cecal ligation and puncture. The vehicle-treated group was given normal saline via intraperitoneal injection (i.p) injection one hour prior to CLP. The bevacizumab-treated group was treated with Bevacizumab at a dose of 0.1mg/kg via i.p injection one hour prior to CLP.

### CLP induction

The CLP procedure was performed as previously described [9]. Mice were anesthetized with ketamine/xylazine, and a midline abdominal incision was made to expose the cecum. The cecum was ligated below the ileocecal valve with a 5.0 suture and double-punctured bilaterally with a 22-gauge needle. After returning the cecum to its anatomical position, the incision was closed with a 6.0 silk surgical suture. Mice were kept in cages and monitored for signs of sepsis every four hours for 24 hours with free access to food and water

### Sample collection

Twenty-four hours after the experiments, the mice were euthanized. Blood samples were collected directly from the heart and allowed to clot at room temperature. Serum was obtained by centrifuging the blood samples at 2,000-3,000 rpm for 20 minutes. These serum samples were then used to measure the levels of alanine transaminase, aspartate transaminase, and angiopoietin-2.

### Histological analysis

Liver specimens were fixed in 10% formalin, washed with normal saline, embedded in paraffin, and cut into 5 µm slices. These slices were stained with hematoxylin and eosin (H&E). An independent pathologist examined the tissues using a light microscope at X100 or X400 magnification. Histopathological changes of the liver tissues were scored from grade 0: no damage; grade 1: mild; grade 2: moderate injury, and grade 3: severe injury and included several features such as vacuoles, focal pyknosis, ballooning, apoptosis, and necrosis [10].

### ELISA assay

Liver specimens were rinsed with a cold phosphate buffer saline (PBS) and weighed for each sample. The PBS containing 1% triton X and 1% protease inhibitor cocktail was added to each sample in a 1:9 W/V ratio. The samples were homogenized using a high-intensity ultrasonic processor and centrifuged for 15 min at 12,000 rpm at 4 °C. The supernatants were transferred into new Eppendorf tubes and used to measure IL-6, TNF-α, MIF, VEGF, ICAM-1, ANGPT2, F2-isoprostane, and caspase-11 according to the manufacturer's instructions

### Statistical analysis

Data were analyzed using GraphPad Prism version 7. Results are expressed as mean±SEM. One-way analysis of variance (ANOVA) followed by the Bonferroni test was used for group comparisons. The Kruskal-Wallis test was applied for histological analysis to compare the degree of tissue damage among groups. A p-value≤0.05 was considered statistically significant.

## RESULTS

### Effect of Bevacizumab on liver function following CLP

**Figure 1:**
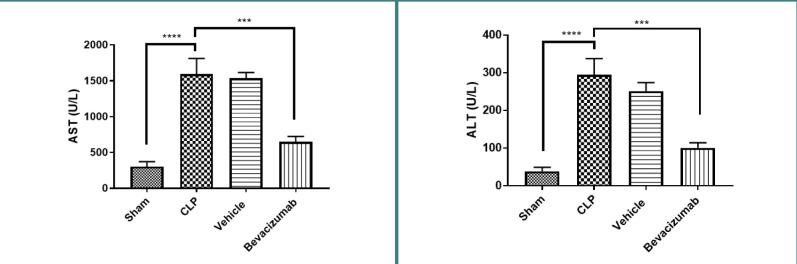
Effect of Bevacizumab on AST and ALT levels following CLP-induced liver injury Serum levels of AST and ALT across the four groups were measured in U/L. Data are presented as mean±SEM, n=6. One-way ANOVA and Bonferroni test.***p≤0.001, ****p≤0.0001

Serum ALT and AST levels were used as indicators of liver injury. Mice exposed to CLP had a significant increase in serum ALT and AST levels compared to the sham group (p≤0.0001). In contrast, treatment with Bevacizumab significantly reduced these levels, suggesting that it could improve liver function exposed to sepsis (Table 1 and 2, Figure 1).

**Table 1:** Mean AST levels among experimental groups measured in U/L

Study groups	AST	p values
Sham group	304.16	
CLP group	1596. 57	p<0.0001 versus sham group
Vehicle-treated group	1538.00	p<0.0001 versus sham group
Bevacizumab-treated group	649.71	p=0.0002 versus CLP

**Table 2:** Mean ALT levels among experimental groups measured in U/L

Study groups	ALT	p values
Sham group	38.16	
CLP group	294.71	p<0.0001 versus sham group
Vehicle-treated group	251.00	p=0.0001 versus sham group
Bevacizumab-treated group	100.57	p=0.0002 versus CLP group

### Effect of Bevacizumab on TNF-α and IL-6 levels following CLP

**Figure 2:**
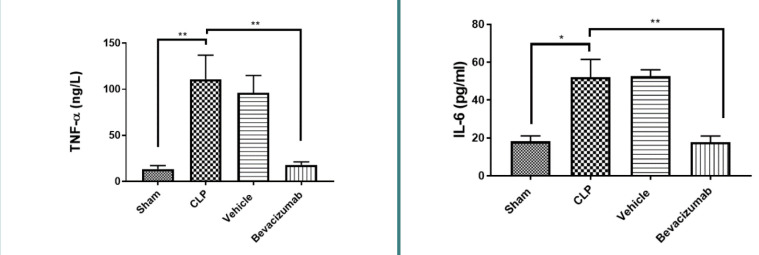
Effect of Bevacizumab on TNF-α and IL-6 levels following CLP Levels of TNF-α and IL-6 in the tissues of the liver across the four groups were measured in ng/L. Results are presented as mean±SEM, n=6. One-way ANOVA and Bonferroni test, **p≤0.01.

TNF-α and IL-6 are well-known cytokines involved in inflammation and key proteins in various cell signaling pathways. Our results showed that mice subjected to CLP had higher levels of TNF-α and IL-6 compared to the sham group (p≤0.01). However, treatment with Bevacizumab led to a significant reduction in these cytokine levels (p≤0.01) (Table 3, Table 4, Figure 2).

**Table 3:** Mean levels of TNF-α among experimental groups measured in ng/L

Study groups	TNF-α	p values
Sham group	13.41	
CLP group	110.86	p=0.0069 versus sham group
Vehicle-treated group	96.20	p=0.0259 versus sham group
Bevacizumab-treated group	17.92	p=0.002 versus CLP group

**Table 4:** Mean levels of IL-6 among experimental groups measured in pg/L

Study groups	IL-6	p values
Sham group	18.26	
CLP group	52.14	p=0.0261 versus sham group
Vehicle-treated group	52.54	p=0.0404 versus sham group
Bevacizumab-treated group	17.83	p=0.0037 versus CLP group

### Effect of Bevacizumab on F2-isoprostane levels following CLP

**Figure 3:**
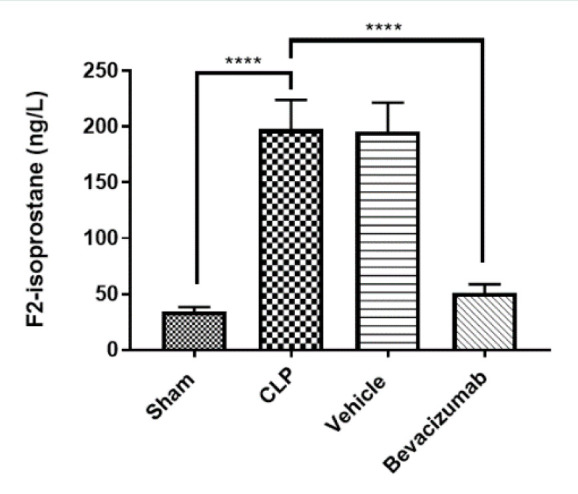
Effect of Bevacizumab on F2-isoprostane levels following CLP The levels of F2-isoprostane in the liver across the four groups were measured in ng/L. Results are presented as mean ±SEM, n=6. One-way ANOVA and Bonferroni test, ***p≤0.001.

F2-isoprostane, a product of lipid peroxidation, is considered a reliable indicator of oxidative stress. Compared to the sham group, the levels of F2-isoprostane in the liver were higher in the CLP group. The Bevacizumab-treated group revealed a significant decrease in the levels of F2-isoprostane (p≤0.001), as shown in Table 5 and Figure 3.

**Table 5:** Mean levels of F2-isoprostane among experimental groups measured in ng/L

Study groups	F2-isoprostane	p values
Sham group	34.36	
CLP group	197.87	p<0.0001 versus sham group
Vehicle-treated group	195.36	p=0.0001 versus sham group
Bevacizumab-treated group	50.96	p<0.0001 versus the control group

### Effect of Bevacizumab on caspases-11 levels following CLP

**Figure 4:**
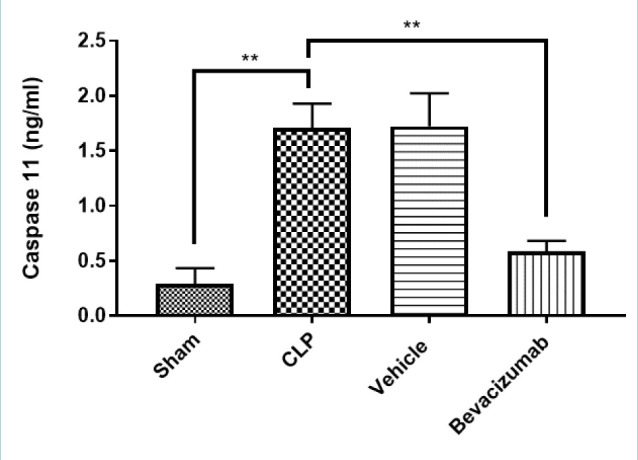
Effect of Bevacizumab on caspase-11 levels following CLP The levels of caspase-11 in the liver across the four groups were measured in ng/L. The results are presented as mean±SEM, n=6. One-way ANOVA and Bonferroni test, **p≤0.01.

The levels of caspase-11 in the CLP group were significantly higher than in the sham group. Conversely, the administration of Bevacizumab reversed these results, leading to a significant reduction in caspase-11 levels in the liver (p≤0.01) (Table 6, Figure 4).

**Table 6:** Mean levels of caspase-11 among experimental groups measured in ng/L

Study groups	Caspases 11	p values
Sham group	0.29	
CLP group	1.70	p=0.0018 versus sham group
Vehicle-treated group	1.72	p=0.0022 versus sham group
Bevacizumab-treated group	0.58	p=0.0036 versus CLP group

### Effect of Bevacizumab on MIF levels following CLP

**Figure 5:**
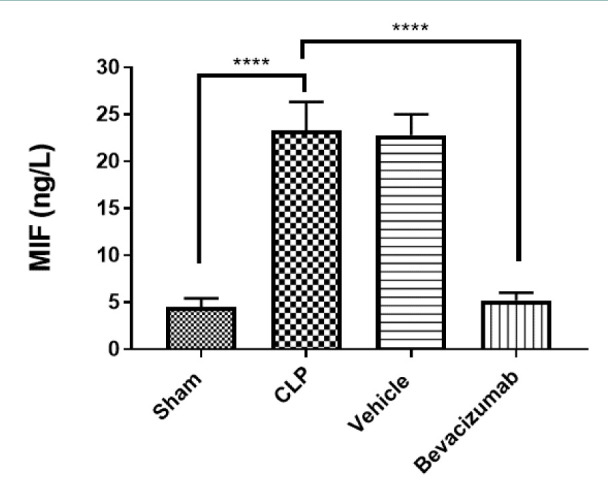
Effect of Bevacizumab on MIF levels following CLP The levels of MIF in the tissues of the liver across the four groups were measured in ng/L. The results are expressed as mean±SEM, n=6. One-way ANOVA and Bonferroni test, ****p≤0.0001.

MIF levels in the liver were markedly elevated in the CLP group compared to the sham group. On the other hand, treatment with Bevacizumab reduced these levels significantly, p≤0.0001 (Table 7, Figure 5).

**Table 7:** Mean levels of MIF among experimental groups measured in ng/L

Study groups	MIF	p values
Sham group	4.45	
CLP group	23.28	p<0.0001 versus sham group
Vehicle-treated group	22.78	p<0.0001 versus sham group
Bevacizumab-treated group	5.22	p<0.0001 versus sham group

### Effect of Bevacizumab on ANGPT2 levels following CLP

**Figure 6:**
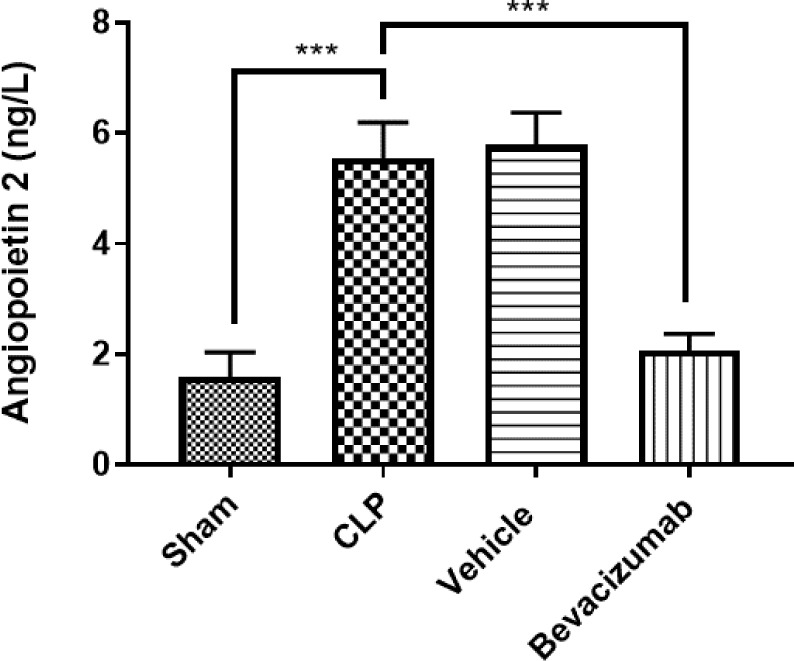
Effect of Bevacizumab on ANGPT2 levels following CLP Serum levels of ANGPT2 across the four groups were measured in ng/L. Data are presented as mean±SEM, n=6. One-way ANOVA and Bonferroni test.***p≤0.001.

Serum levels of ANGPT2 were measured using ELISA assay. The levels of ANGPT2 in the CLP group were ~3.6-fold greater compared to the sham group, p≤0.001. Treatment with Bevacizumab reduced levels of ANGPT2 compared with the CLP group, p≤0.001 (Table 8, Figure 6).

**Table 8:** Mean levels of ANGPT2 among experimental groups measured in ng/L

Study groups	Angiopoietin 2	p values
Sham group	1.59	
CLP group	5.54	p=0.0005 versus sham group
Vehicle-treated group	5.78	p=0.0003 versus sham group
Bevacizumab-treated group	2.06	p=0.0003 versus sham group

### Effect of Bevacizumab on ICAM-1 levels following CLP

**Figure 7:**
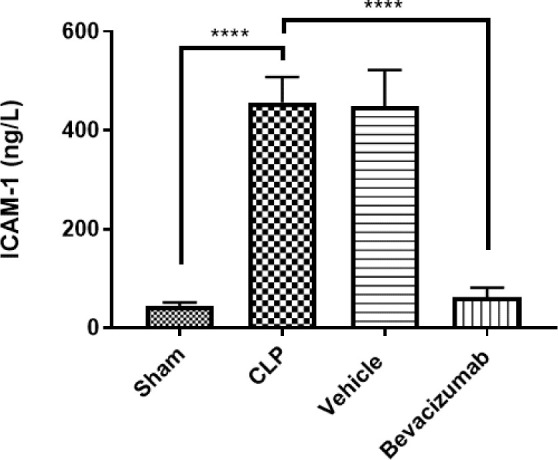
Effect of Bevacizumab on ICAM-1 levels following CLP ICAM-1 levels in the tissues of the liver across the four study groups were measured in ng/L. The results are presented as mean ±SEM, n=6. One-way ANOVA and Bonferroni test, ****p≤0.0001.

Mice subjected to CLP had elevated levels of ICAM-1 in liver tissues. Treatment with Bevacizumab significantly reduced ICAM-1 levels compared to the CLP group (p≤0.001) (Table 9, Figure 7).

**Table 9:** Mean levels of ICAM-1 among experimental groups measured in ng/L

Study groups	ICAM-1	p values
Sham group	43.99	
CLP group	456.71	p<0.0001 versus sham group
Vehicle-treated group	448.80	p<0.0001 versus sham group
Bevacizumab-treated group	62.50	p<0.0001 versus sham group

### Effect of Bevacizumab on VEGF levels following CLP

**Figure 8:**
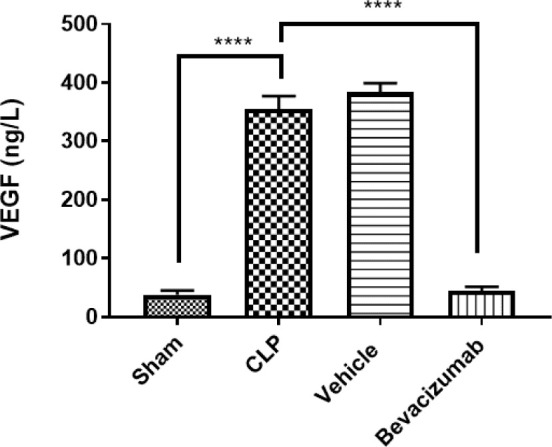
Effect of Bevacizumab on VEGF levels following CLP VEGF levels in liver tissues across the four study groups were measured in ng/L. The results are presented as mean±SEM, n=6. One-way ANOVA and Bonferroni test, ****p≤0.0001.

VEGF, a potent angiogenic signaling protein, is critical in sepsis. In this experiment, the levels of VEGF in liver tissues were measured. The CLP group had a significant increase in VEGF levels compared to the sham group (p≤0.001). In contrast, Bevacizumab treatment resulted in a significant reduction in VEGF levels compared to the CLP group (p≤0.001) (Table 10, Figure 8).

**Table 10:** Mean levels of VEGF among experimental groups measured in ng/L

Study groups	VEGF	p values
Sham group	38.27	
CLP group	354.84	p<0.0001 versus sham group
Vehicle-treated group	383.35	p<0.0001 versus sham group
Bevacizumab-treated group	43.84	p<0.0001 versus CLP group

### Effect of Bevacizumab on liver tissues following CLP

Histological analysis was conducted to assess the severity of liver damage following 24 hours of CLP.

**Figure 9:**
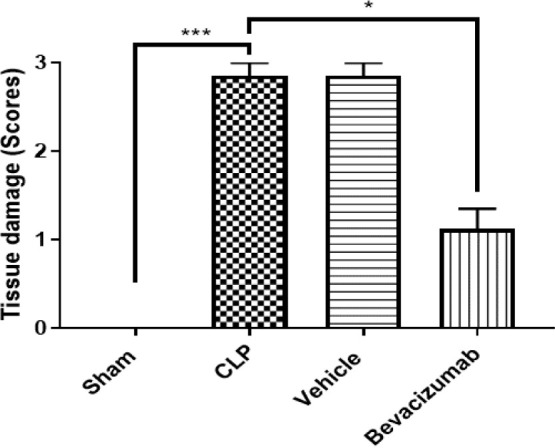
Histological liver damage scores Mean histological scores representing liver damage across four experimental groups. Data presented as mean±SEM, n=6. Kruskal-Wallis test ***p≤0.001.

**Figure 10:**
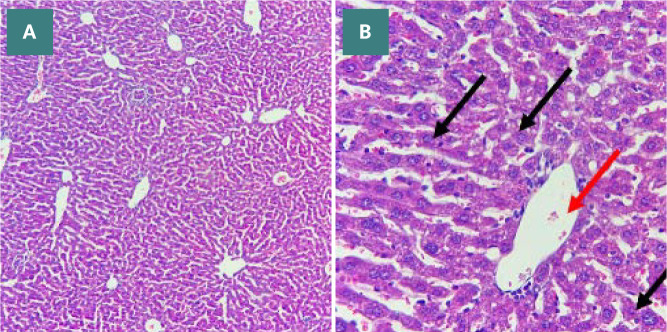
Liver histology in sham group. A: Normal liver histology in the sham group (X 100 magnification). B: Central vein (red arrow) and normal hepatocytes (black arrow) in the sham group (X 400 magnification).

**Figure 11:**
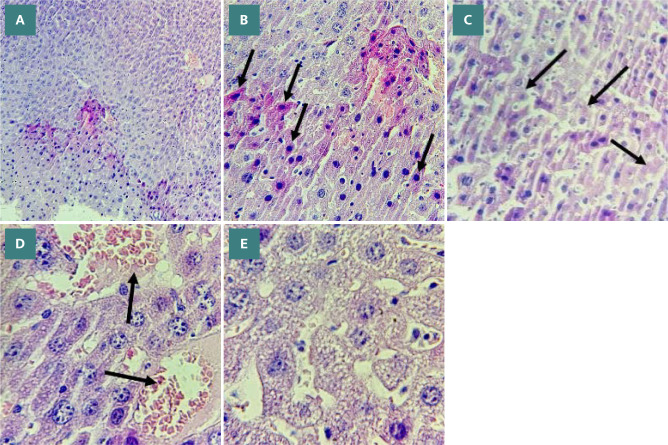
Liver sections showing the morphological changes in the CLP group. A: Severe liver damage in liver sections (score 3) (X100 magnification). B: Cytoplasmic eosinophilia, apoptotic cells with chromatin condensation, pyknotic nuclei (black arrows) (X400 magnification). C: Necrotic cells with nuclear fading (black arrows) (X400 magnification). D: Vascular congestion with erythrocyte stasis (black arrows) (X400 magnification). E: Cytoplasmic vacuoles (X400 magnification).

**Figure 12:**
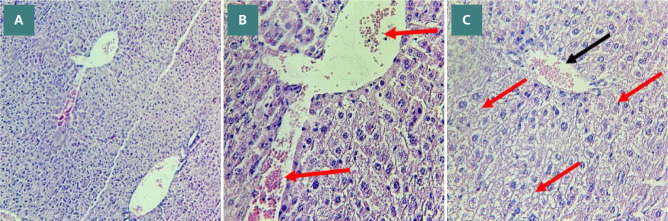
Liver sections showing the impact of Bevacizumab treatment on liver tissues following CLP. A: Mild liver damage in liver sections (score 1) (X100 magnification). B: Vascular congestion with erythrocyte stasis (red arrows) (X400 magnification). C: Vascular congestion with erythrocyte stasis (black arrows), cytoplasmic vacuoles, and steatosis (red arrows) (X400 magnification).

The degree of liver damage was scored: 0, no damage; 1, mild; 2, moderate; 3, severe (Figure 9). Mice in the CLP group revealed a high degree of damage characterized by necrotic cells and nuclear fading compared to the normal liver tissue of the sham group (Figure 10 A-B and 11 A-E). In contrast, the histological analysis of the liver in the Bevacizumab-treated group revealed only mild damage (p≤0.05) (Figure 12 A-C).

## DISCUSSION

Sepsis, a leading cause of severe organ dysfunction resulting from an uncontrolled host response to infection, remains a leading cause of organ dysfunction and mortality among critically ill patients despite advances in antibiotic therapy and resuscitation techniques [11]. The mortality rate among patients with sepsis is around 30% to 50% due to the multi-organ dysfunction and subsequent organ failure. The occurrence of liver dysfunction during sepsis is approximately between 34% and 46%, and liver failure is around 1.3 % to 22% [12]. The mechanisms behind the pathophysiology of organ dysfunction during sepsis are believed to be related to tissue hypoperfusion associated with hypotension, systemic inflammatory response, and diffused intravascular coagulation [13]. The liver is a critical organ and active contributor to various body functions, including the metabolism and the production of several substances involved in different functions. Therefore, protecting the liver could reduce the severity of disease in critically ill patients with sepsis [14].

### Effect of sepsis on liver function parameters (ALS and AST)

The serum levels of ALT and AST, which are main indicators of liver damage, were higher in the CLP and vehicle-treated groups than in the sham groups. These results are consistent with those of Zhu *et al*. [1], who also found high levels of both enzymes in mice subjected to CLP. Furthermore, consistent with our observations, studies conducted in rats exposed to CLP also documented significantly elevated ALT and AST levels compared to the sham group [13]. This result is also consistent with another study that induced liver injury in mice to assess hepatocellular injury 20 hours following CLP and found that serum AST and ALT showed a significant increase compared to the sham group [15]. ALT and AST are released from injured hepatocytes to the circulation. Therefore, elevated levels of AST and ALT are classical laboratory evidence of hepatotoxicity.

### Effect of Bevacizumab on liver function parameters (ALT and AST)

The levels of ALT and AST in the serums were lower in mice treated with Bevacizumab compared to the CLP group. These results align with an earlier study, which revealed that Bevacizumab reduced ALT and AST levels and ameliorated hepatic fibrosis in an animal model of liver fibrosis, suggesting that it could protect from sepsis-induced damage [16].

### Effect of sepsis on IL-6

The levels of IL-6 were significantly increased in both the CLP and vehicle-treated groups compared to the sham group. These findings are consistent with other research, which revealed elevated concentrations of IL-6 in the liver homogenates in CLP and vehicle-treated groups [17]. IL-6 is a pivotal inflammatory cytokine substantially upregulated in sepsis [18]. It is essential to induce acute-phase response and temperature regulation. Several studies highlighted the importance of circulating IL-6 as a beneficial readout in diagnosing bacteremia. Elevated levels of IL-6 in serum correlate with decreased survival rate in patients with sepsis [19].

### Effect of Bevacizumab on IL-6

Treatment with Bevacizumab resulted in a significant reduction in IL-6 levels compared to the CLP group, aligning with recent studies that reported similar decreases in IL-6 levels in mice treated with Bevacizumab following CLP induction [2]. These findings suggest that the blockade of VEGF by Bevacizumab may reduce the production of inflammatory cytokines such as IL-6 and IL-10 [2]. Furthermore, IL-6 levels were decreased in rats treated with subconjunctival injection of Bevacizumab compared to the non-treated group. VEGF acts as a chemoattractant for macrophages and can induce their activation. In this context, Bevacizumab can decrease the release of inflammatory cytokines from activated macrophages [20]. Furthermore, these results agree with previous research conducted in rat models of ulcerative colitis, where concentrations of IL-6 were significantly lower in the Bevacizumab-treated group compared to the control group. This suggests that Bevacizumab does not only affect the VEGF and its receptors but also decreases TGF-β, which is a key mediator of inflammation [21].

### Effect of sepsis on TNF-α

There was a significant increase in TNF-α concentrations in the liver homogenates of the CLP and vehicle-treated groups compared to the sham group. These results are consistent with data obtained in another study, which found that levels of TNF-α were significantly increased in rats subjected to CLP [22]. TNF-α is involved in the stimulation of macrophages, triggering the release of various proinflammatory cytokines such as IL-6, IL-8, reactive oxygen species (ROS), and reactive nitrogen species. These factors can contribute to multi-organ failure, highlighting the central role of TNF-α in the pathophysiology of sepsis. In addition, TNF-α plays a crucial role in inflammation, immunity, apoptosis, and cell survival [23]. Similar results were obtained in another study, which revealed that serum levels of TNF-α significantly increased in the CLP group compared to the control 6 hours after CLP [24], suggesting that inflammatory cytokines such as IL-6 and TNF-α peak at an early phase of sepsis being strongly associated with disease progression. Another study highlighted elevated levels of TNF-α following CLP induction, indicating that sepsis is a crucial condition associated with the overproduction of inflammatory cytokines, which may lead to leukocyte recruitment and tissue damage [1]. TNF-α is a multifunctional cytokine, firstly known as an endogenous soluble factor that stimulates the necrosis of solid tumors. It has become an important pro-inflammatory cytokine involved in the immunopathogenesis of several diseases. TNF-α stimulates a number of biological responses in the liver, such as apoptosis and necroptosis of hepatocytes, inflammation and regeneration of the liver, and autoimmunity [25].

### Effect of Bevacizumab on TNF-α

Treatment with Bevacizumab resulted in a significant decrease in TNF-α levels in liver tissues compared to the CLP group. These results are in line with those of previous reports that investigated the effect of Bevacizumab on acetic acid-induced ulcerative colitis in a rat model and revealed that the levels of TNF-α decreased significantly in the Bevacizumab-treated group. The pro-inflammatory cytokines play a critical role in host defense. However, the overproduction may cause unresolved inflammation and lead to tissue damage. Cytokines contribute to the formation of newly formed blood vessels, which mediate the onset of immune response by supplying the inflamed tissue with oxygen and nutrients [26]. In a traumatic brain injury model, mice treated with Bevacizumab had a significant drop in TNF-α levels, highlighting the ability of Bevacizumab to reduce inflammatory processes and subsequent vascular complications [27]. This anti-inflammatory feature of Bevacizumab was also confirmed in rats subjected to chronic sports arthritic injuries by reducing the levels of TNF-α [28].

### Effect of sepsis on caspase-11

The levels of caspase-11 in the CLP group and the vehicle-treated group were significantly higher than in the sham group. This finding aligns with previous literature reporting increased caspase-11 levels in liver tissues following exposure to lipopolysaccharide (LPS) induction. LPS provokes caspase-11 activation, culminating in pyroptosis, considered the main driver of endotoxic shock. The recent discovery of pyroptosis, a novel form of programmed cell death, has received considerable interest in pharmacology, particularly in relation to caspase-11 targeting [29]. Moreover, increased caspase-11 levels have been documented in a range of tissues following exposure to LPS. For instance, in mice lacking caspase-11, there was a significant reduction in LPS-induced permeability and vascular leakage in lung tissue, which increased survival rates [30]. In mice subjected to LPS, elevated levels of caspase-11 were observed in liver tissues, leading to pyroptosis and the development of chronic liver disease [31].

### Effect of Bevacizumab on caspase-11

The current study showed that Bevacizumab treatment reduced caspase-11 levels in liver tissues compared to the CLP group. To the best of our knowledge, no previous studies have addressed the impact of Bevacizumab on caspase-11 in the context of sepsis-induced liver injury. However, recent studies investigated other apoptotic enzymes in different models, such as caspase-3 and caspase-8. For instance, in a rabbit model of diabetes, intravitreal administration of Bevacizumab resulted in a marked decrease in caspase-3 levels, suggesting that it could have antiapoptotic properties [32].

### Effect of sepsis on ICAM-1

The levels of ICAM-1 in the liver were significantly higher in both the CLP and the vehicle-treated groups than in the sham group. These results are consistent with other findings, which reported a twofold increase in ICAM-1 mRNA levels across various organs, such as the lung, thymus, and spleen, after 24 hours of CLP induction [33]. ICAM-1, an adhesion molecule, plays a pivotal role in regulating the adhesion and migration of neutrophils to target tissues. However, overproduction of ICAM-1 can potentially result in organ dysfunction [33]. Numerous reports highlighted increased levels of ICAM-1 in different organs across many species following CLP. For instance, after 24 hours of CLP, ICAM-1 levels, known for their involvement in neutrophil adhesion to inflamed organs, were significantly higher in liver tissues compared to the sham group [17]. Over the course of 20 hours following CLP induction, a significant increase was observed in the total number of neutrophils expressing ICAM-1 in both the lung and blood cells of mice [34].

### Effect of Bevacizumab on ICAM-1

The most obvious outcome of this study is the significant reduction in ICAM-1 levels observed in the Bevacizumab-treated group compared to the CLP group. These results align with a recent study indicating that Bevacizumab reduced the expression of ICAM-1 in the brain tissue of mice 24 h after traumatic brain injury, suggesting that it could reduce the inflammatory processes induced by traumatic brain injury and prevent the vascular consequences associated with inflammation [27].

### Effect of sepsis on VEGF

There were significant increases in the levels of VEGF in the liver homogenates of the CLP group compared to the sham group. These results align with studies indicating elevated VEGF levels in CLP and LPS-induced endotoxemia models and a positive correlation between sepsis and increased levels of circulating VEGF, with peak levels observed 24 hours after sepsis induction [6]. Researchers have proposed that VEGF may sensitize endothelial cells to the effects of low concentrations of TNF-α, contributing to the increased permeability of endothelial cells—a phenomenon that may be linked to the higher mortality observed in sepsis cases [6]. Furthermore, some reports explored that the level of plasma VEGF increased significantly in mice following CLP compared to the sham-operated group [23].

### Effect of Bevacizumab on VEGF

There was a significant decrease in VEGF levels in the liver of mice treated with Bevacizumab compared to the CLP group. This finding is consistent with Genovese *et al*. [27], who reported a marked reduction in VEGF levels in mice treated with Bevacizumab compared to the control group in a model of traumatic brain injury [27]. Furthermore, Jeong *et al*. [2] reported a similar outcome, suggesting that Bevacizumab acts by binding to two types of receptors: Flt-1 and Flk-1. FIt-1 is located on both endothelial cells and monocytes, while FIk-1 is expressed in endothelial cells, and Bevacizumab possibly inhibits the binding of VEGF to its receptors [2]. In the context of LPS-induced lung injury, the monoclonal antibody Bevacizumab has been shown to inhibit pulmonary vascular permeability [35].

### Effect of sepsis on MIF

The levels of MIF in liver homogenates were higher in mice subjected to CLP than in the sham group. These findings align with the results reported by Tilstam *et al*. [36], who observed a similar increase in MIF levels in peritoneal lavage fluid after 22 hours of CLP procedure compared to the sham group. Furthermore, in a rat model of CLP-induced peritonitis, increased levels of MIF in plasma were found at 6, 20, and 30 h after CLP induction [37]. Moreover, high levels of serum MIF were also found in CLP-induced acute lung injury in rats [38]. Using anti-MIF antibodies to neutralize MIF in vivo reversed the myocardial dysfunction induced by endotoxin in rats [39]. MIF is a crucial factor in the development of sepsis, being involved in the recruitment of inflammatory cells such as macrophages, basophils, neutrophils, and eosinophils to the site of inflammation, resulting in an extensive release of different proinflammatory cytokines such as IL-1β and TNF-α [40].

### Effect of Bevacizumab on MIF

This study found that the levels of MIF were significantly lower in the Bevacizumab-treated group than in the CLP group. These results align with previous studies that reported decreased levels of MIF when treated with Bevacizumab. These findings may be attributed to MIF depletion, likely resulting from reduced VEGF levels and the subsequent loss of MIF transcription mediated by VEGF, a process that Bevacizumab can potentially neutralize. This drug could act indirectly on MIF through its effect on VEGFR2, which may be a critical player in preventing MIF secretion [41].

### Effect of sepsis on F2-isoprostane

CLP induction caused a marked increase in the levels of F2-isoprostane compared to the sham group. F2-isoprostane is a key biomarker for oxidative stress and lipid peroxidation due to its stability and high specificity [42]. These results align with those of Alnfakh *et al*. [43], which also revealed elevated levels of F2-isoprostane in the lung tissue of mice exposed to CLP. Oxidative stress and lipid peroxidation play critical roles in initiating and maintaining the inflammatory response [43]. High levels of F2-isoprostane were detected in the plasma of patients with sepsis, particularly in those with hepatic and renal dysfunction, within two days of onset [42].

### Effect of Bevacizumab on F2-isoprostane

Treatment with Bevacizumab significantly reduced the levels of F2-isoprostane compared to the CLP group. To our knowledge, no prior studies have investigated the effects of Bevacizumab treatment on F2-isoprostane levels in a CLP model. F2-isoprostanes, resulting from lipid peroxidation, are easily detectable in tissues and blood. F2-isoprostanes play a role in different biological functions not only as oxidative molecules but also impact the activation of a variety of proteins, such as thrombin and prostanoid receptors. Therefore, F2-isoprostanes are critical contributors to tissue injury [42].

### Effect of sepsis on angiopoietin 2

The results of this study indicate a significant increase in the levels of ANGPT2 in the serum of mice subjected to CLP compared to the sham group. These findings align with other research that observed a significant increase in plasma levels of ANGPT2 in rats undergoing CLP [44]. ANGPT1 is primarily produced and secreted by cells surrounding the endothelium, whereas ANGPT2 is synthesized by endothelial cells and stored in Weibel-Palade bodies (WPBs). ANGPT2 is quickly released from WPBs when the inflammatory mediator and stimulatory molecules appear [45].

### Effect of Bevacizumab on angiopoietin 2

Treatment with Bevacizumab significantly decreased the levels of ANGPT2 compared to the CLP group. However, research examining the impact of Bevacizumab treatment on ANGPT2 levels is limited, and several questions remain unanswered. It has been reported that patients with advanced metastatic colorectal cancer had high serum levels of ANGPT2, and Bevacizumab therapy reduced these levels, suggesting that it may play a role in modulating its effect on VEGF levels [46]. Further research should be undertaken to explain how Bevacizumab-targeting VEGF could affect the levels of ANGPT2 and the mechanisms involved in this context.

### Effect of CLP on liver tissues

The histological analysis showed that the liver tissues in mice subjected to CLP were characterized by severe morphological damage compared to the sham group. These features included vascular congestion, apoptosis, portal inflammation, and necrosis. These findings align with the research conducted by Li *et al*. [10], which similarly reported severe liver damage as a result of CLP induction, characterized by congestion, cell infiltration, and necrosis. Furthermore, these results are consistent with earlier studies [47, 48].

### Effect of Bevacizumab on liver tissue

Treatment with Bevacizumab improved the morphological features of liver tissues, suggesting its potential role as a protective agent against liver injury. In a brain injury model, Bevacizumab reduced the edema and tissue damage of the brain compared with the non-treated group, which exhibited severe damage and Eosinophilic neurons [27]. Furthermore, in a rat model of hepatic fibrosis induced by carbon tetrachloride (CCl4) administration, Bevacizumab was associated with attenuated liver fibrosis and improved liver function, underscoring its potential as a therapy for liver fibrosis [16]. In addition, in a rat model of acetic acid-induced ulcerative colitis, intraperitoneal injection of Bevacizumab was shown to protect colonic tissues and reduce inflammation damage scores, suggesting that Bevacizumab may hold promise as a future therapeutic option [26].

## CONCLUSION

This study identified that Bevacizumab, a neutralizing monoclonal antibody against VEGF, effectively reduced the levels of proinflammatory cytokines TNF-α, IL-6, MIF, adhesion molecule ICAM-1, and the oxidative stress marker F2-isoprostane. Furthermore, Bevacizumab significantly decreased the levels of angiogenic growth factor VEGF, ANGPT2, apoptotic enzyme, and caspase-11 and mitigated the liver injury following CLP. These findings suggest that Bevacizumab has the potential to attenuate liver injury through its anti-inflammatory, antiangiogenic, and antiapoptotic mechanisms of action.
